# AMPD1: a novel therapeutic target for reversing insulin resistance

**DOI:** 10.1186/1472-6823-14-96

**Published:** 2014-12-15

**Authors:** Jidong Cheng, Hiroko Morisaki, Keiko Toyama, Naomi Sugimoto, Takuya Shintani, Andreas Tandelilin, Tetsuaki Hirase, Edward W Holmes, Takayuki Morisaki

**Affiliations:** Department of Bioscience and Genetics, National Cerebral and Cardiovascular Center Research Institute, 5-7-1 Fujishirodai, Suita, Osaka, 565-8565 Japan; Department of Molecular Pathophysiology, Osaka University Graduate School of Pharmaceutical Sciences, Suita, Osaka, Japan; Sanford Consortium for Regenerative Medicine, San Diego, CA USA; Department of Internal Medicine, The First Affiliated Hospital of Shantou University Medical College, Shantou, Guangdong 515031 P. R. China

**Keywords:** AMP deaminase, Adenine nucleotide, Diabetes, Insulin resistance, AMP kinase, Glucose metabolism

## Abstract

**Background:**

Insulin resistance is one of the hallmark manifestations of obesity and Type II diabetes and reversal of this pathogenic abnormality is an attractive target for new therapies for Type II diabetes. A recent report that metformin, a drug known to reverse insulin resistance, demonstrated in vitro the metformin can inhibit AMP deaminase (AMPD) activity. Skeletal muscle is one of the primary organs contributing to insulin resistance and that the AMPD1 gene is selectively expressed at high levels in skeletal muscle.

**Methods:**

Recognizing the background above, we asked if genetic disruption of the AMPD1 gene might ameliorate the manifestations of insulin resistance. AMPD1 deficient homozygous mice and control mice fed normal chow diet or a high-fat diet, and blood analysis, glucose tolerance test and insulin tolerance test were performed. Also, skeletal muscle metabolism and gene expression including nucleotide levels and activation of AMP activated protein kinase (AMP kinase) were evaluated in both conditions.

**Results:**

Disruption of the AMPD1 gene leads to a less severe state of insulin resistance, improved glucose tolerance and enhanced insulin clearance in mice fed a high fat diet. Given the central role of AMP kinase in insulin action, and its response to changes in AMP concentrations in the cell, we examined the skeletal muscle of the AMPD1 deficient mice and found that they have greater AMP kinase activity as evidenced by higher levels of phosphorylated AMP kinase.

**Conclusions:**

Taken together these data suggest that AMPD may be a new drug target for the reversal of insulin resistance and the treatment of Type II diabetes.

**Electronic supplementary material:**

The online version of this article (doi:10.1186/1472-6823-14-96) contains supplementary material, which is available to authorized users.

## Background

Type II diabetes and the associated disorder referred to as the metabolic syndrome affect more than 380 million people in the world and the prevalence of these disorders is predicted to increase in the future [1]. The fundamental abnormality in glucose metabolism underlying these disorders is insulin resistance [2]. While insulin resistance can be caused by a number of factors, there is a large body of evidence that a high fat diet with resultant fat accumulation in various organs is a major predisposing factor to the development of insulin resistance [2]. One of the predominant organs in which fat accumulates in the setting of a high fat diet is skeletal muscle, and numerous studies have documented that skeletal muscle which is a major site of insulin action contributes to the state of insulin resistance observed in animals and humans consuming a high fat diet [3].

Given that skeletal muscle is one of the major organs that contributes to insulin resistance it stands to reason that therapies directed to targets expressed in this tissue may offer novel approaches to treating these disorders. A recent report demonstrated that AMP deaminase (AMPD) isolated from skeletal muscle is inhibited by metformin, one of the best known drugs for reversing the pathologic state of insulin resistance [4]. While the results of this study are intriguing it is difficult to assess if inhibition of AMPD is the target responsible for the in vivo effects of this drug on insulin action since many studies have reported that metformin affects the activity of numerous enzymatic activities [5].

To address the question of whether AMPD represents a valid new target for drug development to reverse or ameliorate insulin resistance we have taken the approach of gene disruption to test this hypothesis more definitively. In particular we elected to reduce the level of AMPD enzyme activity in skeletal muscle through disruption of the AMPD1 gene, the predominant AMPD gene expressed in skeletal muscle [6, 7]. The results described in this report demonstrate that mice rendered deficient in AMPD1 enzyme activity have a milder state of insulin resistance, improved glucose tolerance, and enhanced insulin clearance when fed a high fat diet when compared to controls with normal skeletal muscle AMPD enzyme activity.

## Methods

### Mice

We previously described the generation of AMPD1 deficient mice [7]. Heterozygous AMPD1 deficient mice (A1(+/−) mice) were backcrossed with C57BL/6 control mice more than 10 times. Genotyping was performed as described before [7]. Animals had free access to food (CE-2, CLEAR) and water, and were housed in a controlled SPF environment with a 12-h light–dark cycle and constant temperature (25°C).

AMPD1 deficient homozygous mice (A1(−/−) mice) and control mice (Wt mice) at 5 weeks old were fed normal chow diet (CD: CE-2) or a high-fat diet (HFD: HFD32) (CLEAR, Tokyo, Japan) with 60% fat calories for 12 weeks.

All animal experiments were approved by the Committee on Animal Research of National Cerebral and Cardiovascular Center and were performed according to the guidelines for the protection of experimental animals of National Cerebral and Cardiovascular Center.

### CT scan

The adiposity of mice was examined radiographically using CT (LaTheta LCT-100, ALOKA) according to the manufacturer’s protocol. Two-mm intervals were scanned from the diaphragm to bottom of the abdominal cavity.

### Blood analysis: insulin, FFA, triglyceride, cholesterol, adiponectin and leptin

Samples were obtained from the mice fasted for 17 h. Plasma insulin levels were measured using an ELISA kit (Morinaga, Yokohama, Japan). Serum FFA, triglyceride, cholesterol were detected by ACS-ACOD methods, GPO-HDAOS methods and HDAOS methods. The levels of leptin, adiponectin were measured using a mouse leptin ELISA kit (Morinaga, Yokohama, Japan), a mouse adiponectin ELISA kit (Ootuka, Tokyo, Japan), respectively.

### Glucose and insulin tolerance

Groups of male mice before and after HFD were used for intraperitoneal glucose injection. After a 17-hr fast, plasma insulin and glucose levels were measured and d-glucose (2 g/kg body weight) was administered. Blood samples were taken from the tail vein at indicated times. Blood glucose levels were detected at 15, 30, 60, 90, 120 min and blood glucose concentration was determined using a glucometer.

Groups of male mice before and after HFD were used for insulin tolerance tests. Mice were fasted for 5 h. Blood glucose levels were detected at 0, 15, 30, 60, 90 min after intraperitoneal injection 0.75 units insulin/kg body weight.

### Western blot analysis

Tissues were sonicated and homogenized in RIPA buffer (1× PBS, 1% Nonidet P-40, 0.5% sodium deoxycholate, 0.1% SDS, 100 μg/ml phenylmethylsulforyl fluoride, 45 μg/ml aprotinin, 100 mM sodium orthovanadate). The supernatant of the homogenate was used for protein determination with a BCA Protein Assay Kit (Pierce, IL, USA) and electrophoresis. Samples were loaded on SDS-PAGE gel with the same amount of total protein. Electrophoretic transfer to polyvinylidene difluoride membranes was followed by immunoblotting with different primary antibodies. Signals were developed with an ECL kit (Amersham, Piscataway, USA).

## Results

### Glucose and insulin dynamics

**Figure 1:**
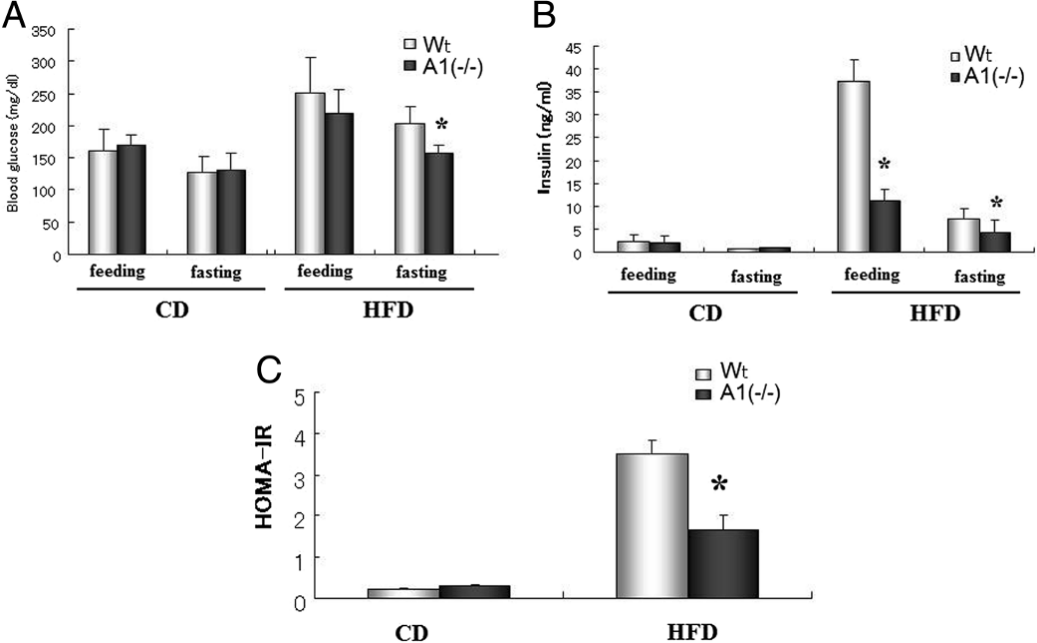
**Blood glucose and insulin levels. A**. Blood glucose levels in mice fed with normal chow diet (CD) and after high fat diet (HFD) challenge. **B**. Blood insulin levels in mice fed with CD and after HFD challenge. C. HOMA-IR index in mice fed with CD and after HFD challenge. Wt: wild type mice, A1(−/−): AMPD1 deficient homozygote mice. *: significant difference between Wt and A1(−/−) mice.

To test the basic premise of this study, i.e. does a loss of AMPD activity alter insulin resistance, we examined blood glucose and insulin levels in wild-type and A1(−/−) mice fed a standard chow diet (CD) and a high fat diet (HFD). As shown in Figure 1, after 12 weeks on a HFD A1(−/−) mice had a significantly lower fasting blood glucose than the wild-type mice and blood insulin levels were significantly lower in the A1(−/−) mice on the HFD in both the fasting state and 3 hours after re-feeding. HOMA-IR analysis documented that insulin resistance was reduced in the A1(−/−) mice relative to the wild-type mice on the HFD.

**Figure 2:**
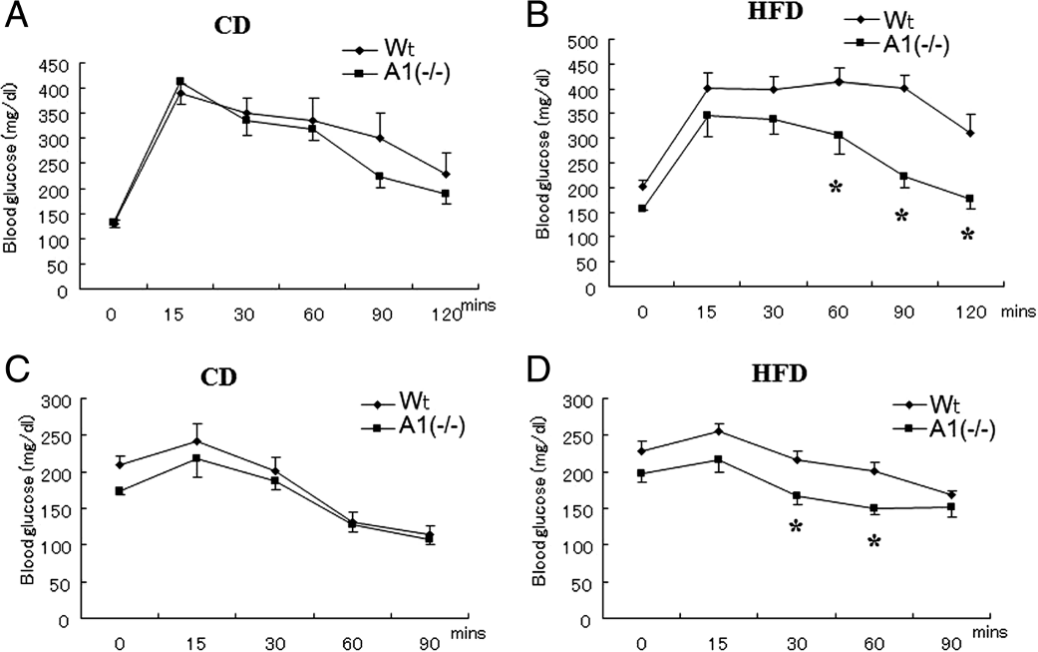
**Glucose tolerance test and insulin tolerance test. A**. Glucose tolerance test in mice fed with normal chow diet (CD). **B**. Glucose tolerance test in mice after high fat diet (HFD) challenge. **C**. Insulin tolerance test in mice fed with normal chow diet (CD). **D**. Insulin tolerance test in mice fed with normal chow diet (CD). Wt: wild type mice, A1(−/−): AMPD1 deficient homozygote mice. *: significant difference between Wt and A1(−/−) mice.

Glucose and insulin dynamics were further assessed with glucose and insulin tolerance tests in these mice (Figure 2). Figure 2A demonstrates that blood glucose levels were not appreciably different in the wild-type and A1(−/−) mice on a standard chow diet after a glucose challenge. In contrast, Figure 2B shows the A1(−/−) mice maintained on a HFD had a more rapid return of blood glucose levels to normal levels than did the wild type animals following a glucose challenge. Figure 2C and D illustrate the results of the insulin tolerance test in these two groups of mice. The A1(−/−) mice maintained on a HFD had significantly lower blood glucose levels 30 and 60 min after insulin administration.

**Figure 3:**
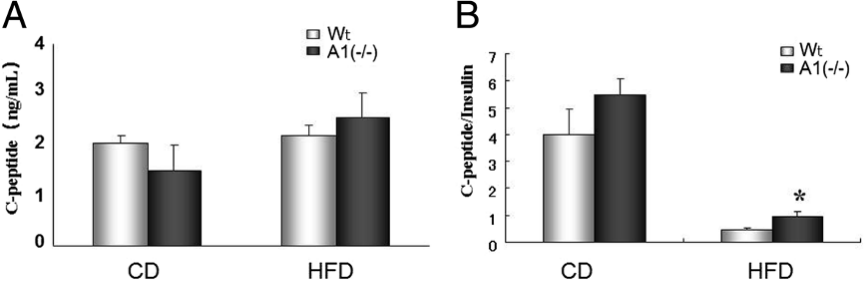
**Insulin production. A**. C-peptide levels in mice fed with normal chow diet (CD) and after high fat diet (HFD) challenge. **B**. C-peptide/Insulin ratios in mice fed with normal chow diet (CD) and after high fat diet (HFD) challenge. Wt: wild type mice, A1(−/−): AMPD1 deficient homozygote mice. *: significant difference between Wt and A1(−/−) mice.

Prior studies have demonstrated insulin clearance is reduced in obese, hyperinsulinemic human subjects [8], and a recent study has indicated insulin clearance is associated with AMPD1 haplotype in man [9]. To assess insulin clearance in the A1(−/−) animals we determined insulin levels relative to insulin production as measured by C-peptide levels. As shown in Figure 3, the ratio of C-peptide/insulin was significantly reduced in both groups of animals on a HFD, though the reduction in the ratio was significantly lower in the A1(−/−) mice than in Wt mice.

### Body weight and adiposity

**Figure 4:**
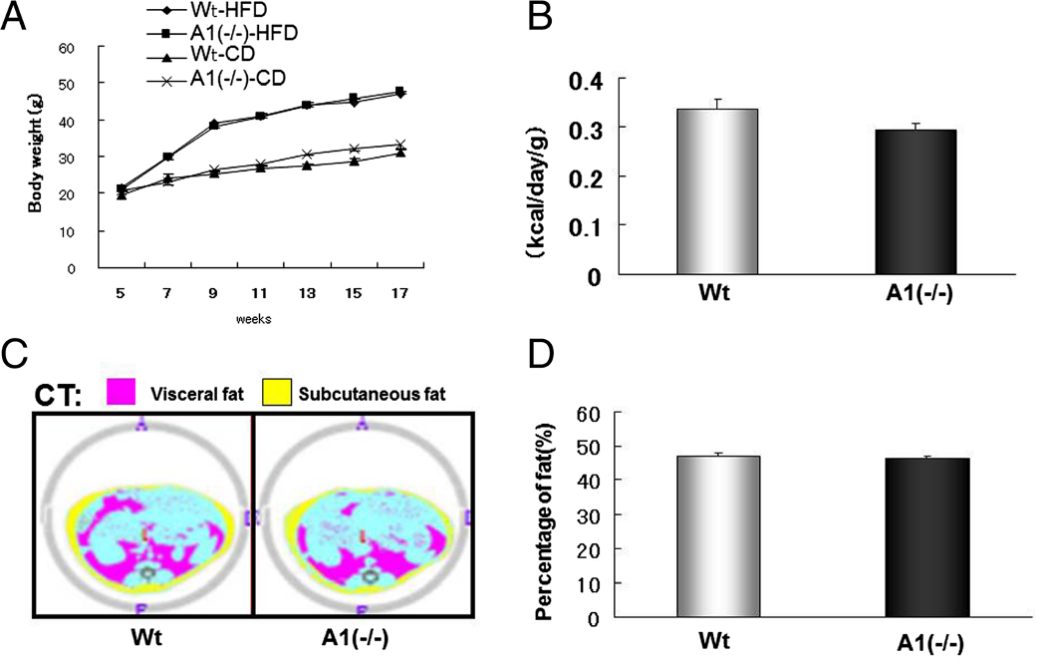
**Body weight and fat accumulation change after high fat diet (HFD) challenge. A**. Body weight change. Wt-CD: wild type mice fed with normal chow diet, A1(−/−)-CD: AMPD1 deficient homozygote mice fed with normal chow diet, Wt-HFD: wild type mice after HFD challenge, A(−/−)-HFD: AMPD1 deficient homozygote mice after HFD challenge. **B**. Food intake during HFD challenge. **C**. CT scan. **D**. Fat ratio calculated by the CT scan exam. Wt: wild type mice, A(−/−): AMPD1 deficient homozygote mice.

Both wild-type and A1(−/−) mice gained weight on the HFD relative to the cohorts fed CD but there was no difference in the incremental weight gain between the Wt and A1(−/−) mice on a HFD (Figure 4A). Food consumption was comparable in the two groups of mice on a HFD (Figure 4B). Total body fat was assessed by CT scans, and while both groups of mice accumulated more fat on the HFD relative to those on the standard CD, consistent with the greater rate of weight gain on the HFD, there was no detectable difference in total body fat relative to total body weight in the A1(−/−) mice compared to Wt mice (Figure 4C and D). Because of the central role skeletal muscle plays in insulin resistance [3] and because AMPD1 is predominately expressed in this tissue, fat content of the skeletal muscle was assessed. As with total body fat, skeletal muscle fat increased in both groups of mice while on the HFD (data not shown) but there was no obvious difference between the A1(−/−) and wild-type mice on HFD (Additional file 1: Figure S1).

Blood lipids and lipid-related hormones were quantitated in both groups of mice on both the CD (data not shown) and the HFD (Table 1). None of these parameters of lipid metabolism was appreciably different between the A1(−/−) and wild-type mice on either diet.

**Table 1:** **The parameters of lipid metabolism in blood**

	Wt	A1(−/−)
TG (mg/dl)	41 ± 8	42 ± 3
FFA (mEq/L)	512 ± 95	473 ± 63
TC (mg/dl)	271 ± 11	240 ± 22
Leptin (ng/ml)	50 ± 4	43 ± 3
Adiponectin (mg/L)	15 ± 4	15 ± 5

n = 5.

Wt: control mice; A1(−/−): AMPD1 deficient homozygous mice. Fasting for 17 hrs after 12 weeks HFD challenge. TG: triglyceride, FFA: free fatty acid, TC: total cholesterol.

### Skeletal muscle metabolism and gene expression

Gastrocnemius muscle of sedentary animals was analyzed for purine metabolites in A1(−/−) and Wt mice on a HFD (Table 2) (ATP, ADP, AMP, IMP, adenosine and cAMP). ATP, ADP, AMP, adenosine, and cAMP levels were not detectably different in skeletal muscle of the two groups of mice. However, IMP was undetectable in the skeletal muscle of A1(−/−) mice in contrast to the easily detectable levels in Wt mice.

**Figure 5:**
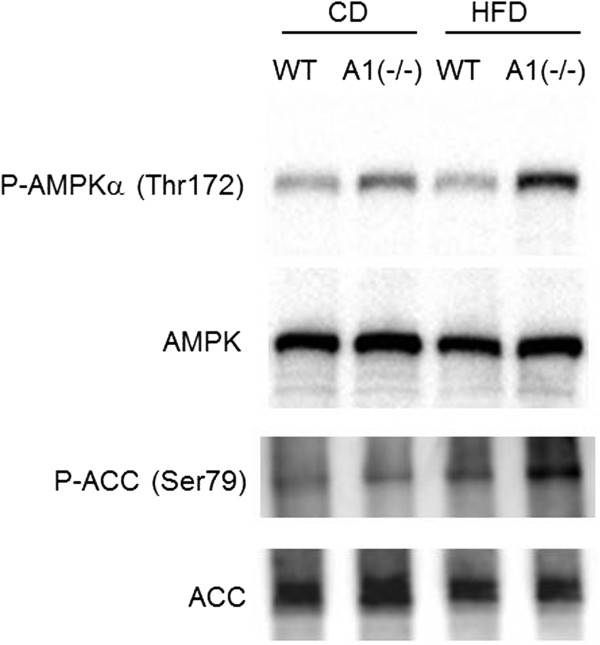
**Western blot study for phosphorylation of AMPK and ACC.** Phosphorylation of AMPK and ACC in skeletal muscles. Antibody for phosphorylated AMPK, AMPK, ACC, or phosphorylated ACC was used. P-AMPKα (Thr172): phosphorylated AMPK at Thr172, AMPK: total AMPK. P-ACC (Ser79): phosphorylated ACC at Ser79. ACC: total ACC. Wt: wild type mice, A(−/−): AMPD1 deficient homozygote mice. CD: fed with normal chow diet, HFD: after high fat diet challenge.

A potential consequence of loss of AMPD activity in skeletal muscle, and a potential explanation for the observed changes in insulin sensitivity, might be differential activation of AMP activated protein kinase (AMPK) in the A1(−/−) mice. As shown in Figure 5, phosphorylation of AMPK was greater in gastrocnemius muscle of A1(−/−) than in Wt animals fed the standard CD. Upon HFD challenge, we observed an even greater increase in phosphorylation of AMPK in gastrocnemius muscle of A1(−/−) compared with Wt animals. Also, acetyl-CoA carboxylase (ACC) was similarly phosphorylated in A1(−/−) mice and this change was prominent after HFD challenge.

**Table 2:** **Nucleotides, adenosine and cAMP in skeletal muscles**

	Wt	A1(−/−)
ATP (nmol/mg)	10.00 ± 0.25	9.90 ± 0.28
ADP (nmol/mg)	2.79 ± 0.13	2.78 ± 0.13
AMP (nmol/mg)	0.36 ± 0.01	0.40 ± 0.03
IMP (nmol/mg)	0.26 ± 0.04	not detected
Adenosine (pmol/mg)	9.54 ± 0.71	9.86 ± 0.44
cAMP (pmol/mg)	1.00 ± 0.10	0.99 ± 0.09

mean ± SE (nmol or pmol/mg wet weight), n = 5.

**Figure 6:**
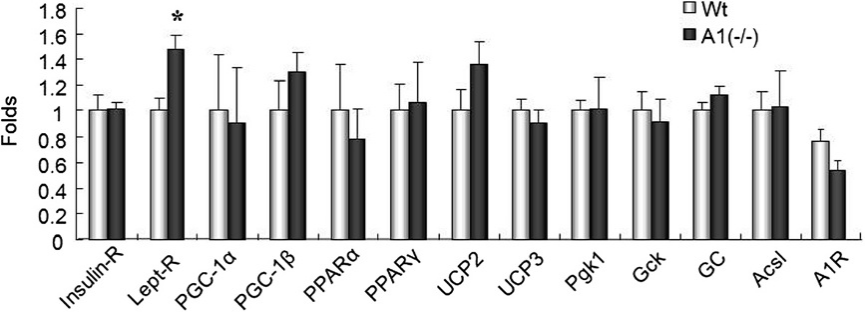
**mRNA expression of various molecules after high fat diet challenge.** Insulin-R: insulin receptor, Lept-R : reptin receptor, Pgk1:phosphoglyceratekinase 1, Gck: glucokinase, Ascl: acyl-CoA synthetase, A1R: adenosine type 1 receptor. Wt: wild type mice, A(−/−): AMPD1 deficient homozygote mice. CD: fed with normal chow diet, HFD: after high fat diet challenge. *: significant difference between Wt and A1(−/−) mice.

Expression of a number of genes has been associated with changes in insulin sensitivity [10]. A limited survey of these genes, as judged by mRNA levels, revealed no significant differences in expression of any of these genes with one exception (Figure 6). Leptin receptor mRNA was about 50% more abundant in skeletal muscle of the A1(−/−) mice.

## Discussion

The results of this study demonstrate that disruption of the AMPD1 gene with reduction of AMPD activity in skeletal muscle protects mice from a number of the adverse metabolic consequences of a high fat diet. The following indices of glucose metabolism are significantly improved in A1(−/−) mice on a high fat diet: fasting blood glucose is lower, fasting and post-prandial insulin levels are lower, glucose and insulin tolerance tests are improved, and insulin resistance is less pronounced in the A1(−/−) animals. In addition to these measures of insulin action A1(−/−) animals exhibited a higher rate of insulin clearance on a high fat diet. While insulin clearance is determined by many factors including renal excretion it is also influenced by insulin uptake by peripheral organs such as skeletal muscle [3].

These studies do not indicate which organ(s) are responsible for these various measures of enhanced insulin action but given the central importance of skeletal muscle in contributing to the state of insulin resistance in animals and humans on a high fat diet [3, 11] and recognizing that AMPD1 is only highly expressed in skeletal muscle of humans and rodents [6], it points to changes in skeletal muscle metabolism as the most likely tissue to produce the observed changes in insulin and glucose metabolism in the whole animal.

As expected, fat content of skeletal muscle increased in mice on a high fat diet, as did fat content in other tissues. But there were no demonstrable differences in fat content of skeletal muscle, or fat content of other tissues, in the A1(−/−) animals, suggesting that the mechanism for enhanced insulin sensitivity is not the result of reduced levels of fat in skeletal muscle or other organs. Consistent with these findings A1(−/−) mice exhibited a comparable increment in weight gain resultant from the high fat diet.

Many interventions and drugs have been reported to improve insulin sensitivity through affecting the activity of AMP kinase [5]. Reduction of AMPD activity with resultant alternations in adenine nucleotide metabolism might be expected to lead to activation AMP kinase which is known to be regulated by adenine nucleotides [5]. Consistent with this hypothesis we observed an increase in the levels of phosphorylated AMP kinase in skeletal muscle of A1(−/−) mice. In sedentary animals we did not detect any changes in the total concentration of AMP or any other adenine nucleotides but we were not able to quantitate the levels of free AMP, the most critical effector of AMP kinase activation, and we postulate that the small fraction of total AMP that is in the pool of free AMP may have been increased leading to the activation of AMP kinase. This conclusion is supported by the failure to detect any IMP, a product of the AMPD reaction, in the skeletal muscle of the A1(−/−) animals.

An unexpected and potentially interesting finding with regard to the phenotypic changes observed in AMPD deficient mice is the increase in expression of the leptin receptor in the skeletal muscle of these animals. Leptin is known to increase the amount phosphorylated AMP kinase in skeletal muscle [12] and it is possible that enhanced activity of the leptin pathway may also have contributed to the observed increase in phosphorylated AMP kinase in the skeletal muscle of the A1(−/−) mice. How alterations in AMPD activity affect expression of the leptin receptor remain to be determined but this observation suggests that alterations in AMPD activity may lead to changes in other pathways that affect insulin and glucose metabolism in myocytes. In this regards, the crossing A1(−/−) mice with db/db mice will provide the further evidence for the interaction between AMPD activity and the leptin receptor.

Decreased levels of IMP in skeletal muscles in A1(−/−) mice indicated that other AMPD genes were not upregulated in muscles. In fact, total AMPD activity was quite low in muscles of A1(−/−) mice as reported before [7]. Therefore, skeletal muscles in A1(−/−) mice should have striking difference in metabolic conditions if there were any change of endogenous AMPD1 at baseline and after 12-week HFD in Wt mice. Also, besides the skeletal muscle, liver, adipose tissue, and others have an important role in regulating glucose metabolism at baseline and after HFD challenge. However, we found that no difference of AMPD2 mRNA expression was observed in liver between Wt and A1(−/−) mice at base line or after HFD challenge (Additional file 2: Figure S2), while AMPD2 mRNA expression was slightly increased in liver of both mice after HFD. Therefore, we thought that the AMPK activity at baseline or after HFD challenge in other organs was not appreciably affected by AMPD1 deficiency.

Regarding AMPK activation in A1(−/−) mice, it could be possible that other metabolites including IMP, hypoxanthine, or uric acid in the pathway downstream to AMPD might play a role for it. Further investigation will be awaited for delineation of precise mechanisms for AMPK activation in AMPD1 deficiency.

During the revision of this report, other reports indicated that AMPD1 deficient mice showed only moderate increase in AMPK activation despite increase in AMP levels and AMP/ATP ratio and that there were no favorable metabolic phenotype in AMPD1 deficient mice after HFD challenge [13, 14]. We think that different protocol for HFD challenge created different results and that further study is awaited for delineating the importance of AMPD1 on insulin metabolism.

## Conclusions

In conclusion, the results of this study validate AMP deaminase as potential new drug target for the amelioration of insulin resistance, which is one of the underlying causes of the metabolic syndrome and Type II diabetes. The results indicate that inhibition of AMPD may lead to improved insulin dynamics in the resting state and without the requirement of concomitant weight loss or reduction in fat content of skeletal muscle. While the mechanism(s) by which reductions in AMPD activity lead to this beneficial metabolic profile need more study the data are consistent with activation of AMP kinase, a known pathway for decreasing insulin resistance, and potentially a novel mechanism of action that may involve the leptin receptor. The effects of reduced AMPD activity in altering insulin action may not be unique to the mouse as two studies have reported that AMPD1 deficient human subjects have a lower incidence of type II diabetes [15, 16], an intriguing result that needs to be confirmed in a larger group of individuals, and the demonstration that AMPD1 haplotype is associated with changes in insulin clearance in man [9]. AMPD1 deficiency is one of the most common inherited defects in the Caucasian population, with approximately 2% of individuals in this ethnic group being homozygous for this gene defect, and few if any of these individuals experience any adverse effects from this enzyme deficiency [6] suggesting that not only would inhibition of AMPD have beneficial effects in patients with the metabolic syndrome and diabetes but inhibition of this enzyme may lead to few deleterious effects.

## Electronic supplementary material

Additional file 1: Figure S1: Sudan Black staining of skeletal muscles. Wt: wild type mice, A(−/−): AMPD1 deficient homozygote mice. (JPEG 56 KB)

Additional file 2: Figure S2: AMPD2 mRNA expression after high fat diet challenge. Wt: wild type mice, A(−/−): AMPD1 deficient homozygote mice. CD: fed with normal chow diet, HFD: after high fat diet challenge. (JPEG 17 KB)

## Abbreviations

AMP: Adenine monophosphate
ADP: Adenine diphosphate
ATP: Adenine triphosphate
AMPD: AMP deaminase
AMP kinase: AMPK: AMP activated protein kinase
ACC: Acetyl-CoA carboxylase
A1: AMPD1
Wt: Control wild type
CD: Chow diet
HFD: High fat diet
IMP: Inosine monophosphate
cAMP: cyclic AMP.
