# Supplementary figures and images for: AMPD1: a novel therapeutic target for reversing insulin resistance
Source: BMC Endocr Disord. 2014 Dec 15;14:96. doi: 10.1186/1472-6823-14-96 (PMC4274759; doi:10.1186/1472-6823-14-96)

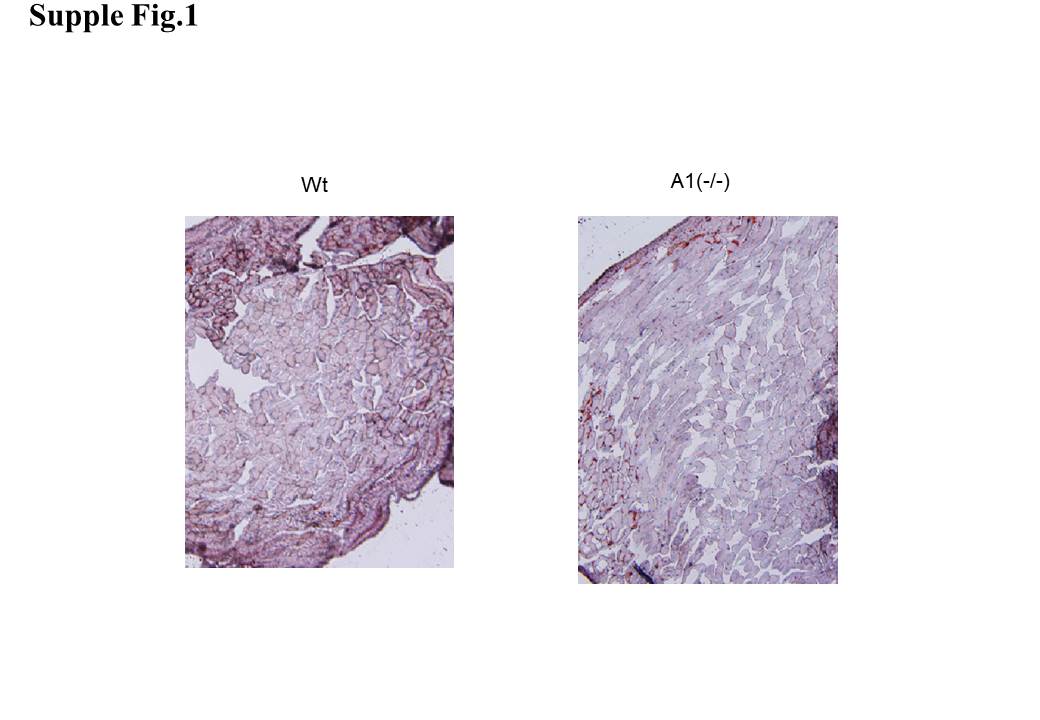

Supplement: Supplementary file 1 — Additional file 1: Figure S1: Sudan Black staining of skeletal muscles. Wt: wild type mice, A(−/−): AMPD1 deficient homozygote mice. (JPEG 56 KB) [file 12902_2014_295_MOESM1_ESM.jpeg]

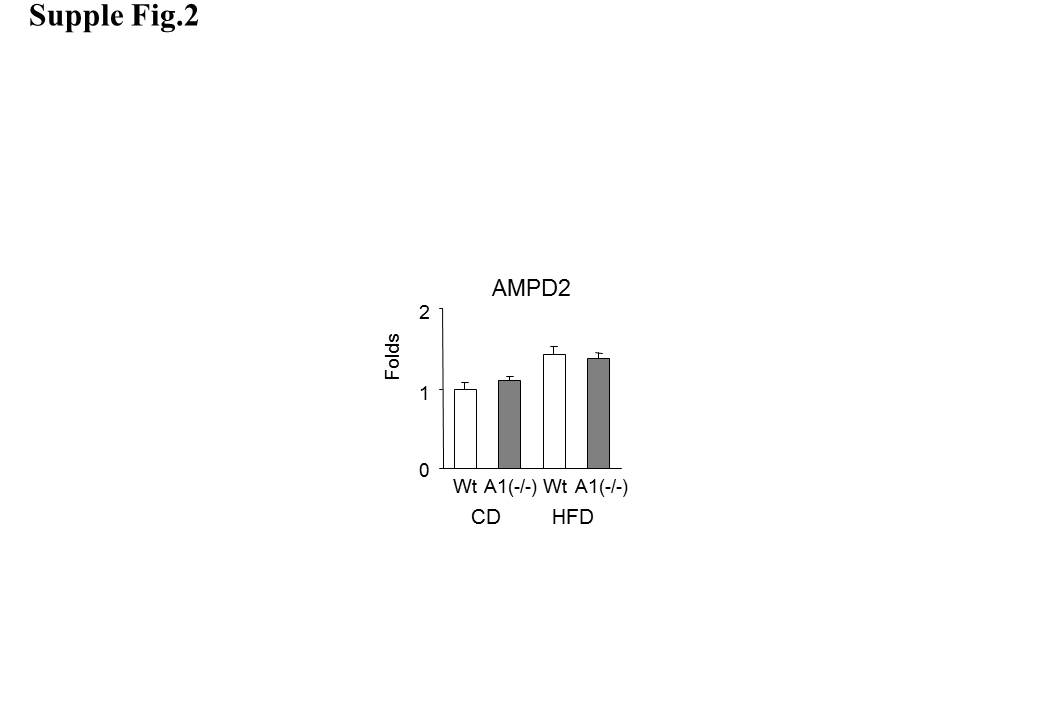

Supplement: Supplementary file 2 — Additional file 2: Figure S2: AMPD2 mRNA expression after high fat diet challenge. Wt: wild type mice, A(−/−): AMPD1 deficient homozygote mice. CD: fed with normal chow diet, HFD: after high fat diet challenge. (JPEG 17 KB) [file 12902_2014_295_MOESM2_ESM.jpeg]
